# Evaluation of a short, interactive diabetes self-management program by pharmacists for type 2 diabetes

**DOI:** 10.1186/s13104-018-3952-y

**Published:** 2018-11-26

**Authors:** Renu F. Singh, Panteha Kelly, Alexander Tam, Jason Bronner, Candis M. Morello, Jan D. Hirsch

**Affiliations:** 1 Skaggs School of Pharmacy and Pharmaceutical Sciences, University of California, San Diego, 9500 Gilman Drive, La Jolla, CA 92093-0657 USA; 2 Department of Pharmacy, UC San Diego Health, 9435 Medical Center Drive, MC 7727, La Jolla, CA 92037 USA; 3 Present Address: 9500 Gilman Drive, MC 0764, La Jolla, CA 92093 USA; 4 Present Address: AIDS Healthcare Foundation, 4071 18th St., San Francisco, CA 94116 USA; 5 UC San Diego Health Internal Medicine, 8939 Villa La Jolla Drive, La Jolla, CA 92037 USA; 6 Present Address: St Luke’s Internal Medicine, 4800 N Cloverdale Road, Boise, ID 82713 USA

**Keywords:** Diabetes, Self-management, Education, Pharmacist

## Abstract

**Objective:**

Numerous barriers prevent patients with type 2 diabetes (T2D) from completing a diabetes self-management program. We investigated whether patients with T2D exhibited improved clinical outcomes after attending a relatively short, interactive diabetes self-management program conducted by pharmacist diabetes educators, compared to a physician’s usual care.

**Results:**

We retrospectively analyzed the data of adults with T2D who attended a diabetes self-management program (≥ 1 group meeting or individual appointment followed by a telephone interview from a pharmacist diabetes educator between May 2010 and Dec. 2012; n = 513) and compared their outcomes with those of T2D patients who received only their physician’s usual care (n = 857). Each patient’s A1C was assessed at baseline, 3 months, and 6 months post-intervention. The mean [SD] reduction in A1C percentage points in the T2D patients was significantly greater in the diabetes self-management program group compared to the physician’s usual care group at both 3 months (− 0.8% [1.5] vs. − 0.2% [0.9], p < 0.001) and 6 months post-intervention (− 0.6% [1.3] vs. − 0.2% [1.1], p < 0.001). T2D patients significantly improved their glycemic control within 3–6 months of attending the diabetes self-management program compared to patients who only received their physician’s usual care.

## Introduction

Diabetes self-management education (DSME) programs educate and empower patients with diabetes to improve their lifestyles and self-coping skills. A 2015 national survey of diabetes educators in the United States reported that < 25% of patients with diabetes receive DSME within the first year of their diagnosis, and this value was unchanged from a survey conducted 3 years earlier [1, 2]. The survey also revealed that of diabetes patients who do enroll in a DSME program, < 30% complete more than 75% of the program [2], suggesting that there are barriers to completing DSME programs in their entirety.

In 2010, two pharmacist diabetes educators (authors RS and PK) initiated the diabetes self-management program (DSMP) within three University of California San Diego Health System (UCSDHS) outpatient clinics. Recognizing that patients often have limited time to attend an educational series, this unique program was designed to offer a relatively brief but highly interactive format. We then evaluated the effectiveness of the program by comparing the mean change in A1C at 3 months post-intervention between patients with type 2 diabetes (T2D) who attended this DSMP and T2D patients who received usual care from their primary care physician (UC). Our secondary aims were to compare (1) the change in A1C between the DSMP and UC groups at 6 months post-intervention, (2) the change in body mass index (BMI) between the DSMP and UC groups at 3 and 6 months from baseline, and (3) the percentage of patients who underwent an eye examination or foot examination within 12 months of participating in the DSMP compared to the percentage in the patients who received only UC.

## Main text

### Methods

#### Study design

This was a retrospective, observational, cohort study with a non-randomized comparator group.

#### Study population

Data for patients ≥ 18 years old with T2D who had attended at least one group meeting or one individual appointment in the DSMP from May 1, 2010 through December 31, 2012 were analyzed. UC patients’ data were obtained by querying the electronic medical records (EMR) system database for a random sample of patients ≥ 18 years old with the diagnosis of T2D who were seen by a primary care physician during the same time period, but who did not enroll in the DSMP.

#### Intervention

Adult patients with diabetes were referred to the DSMP for group meetings or individual appointments by their healthcare providers at UCSDHS, based on the patients’ data in their EMR. Conducted by a pharmacist diabetes educator, the group meetings consisted of two core sessions, each 2 h in length, covering 3–4 diabetes self-management topics per session and spaced 1–2 weeks apart. The DSMP was conducted on weekdays, with group meetings and individual appointments offered during the day or evening. Prior to each class, the pharmacist diabetes educator reviewed each patient’s medical record, including his or her diabetes medications, glycemic control, and reason for referral. This enabled the educator to tailor the group class to the needs of the individual patients to a certain degree. All DSMP patients received a telephone follow-up call from a pharmacist educator 1–2 weeks after their completion of the final group meeting or individual appointment.

The diabetes pharmacist educators practiced under a limited collaborative practice agreement for the DSMP that enabled them to order diabetes testing supplies, refills of existing diabetes medications, pertinent laboratory tests, and referrals for annual eye exams.

#### Outcome measures

This study measured the mean change in A1C and BMI at baseline compared to 3 months and 6 months post intervention between the DSMP and UC groups. The closest A1C and BMI values within 6 months prior to the date of the first DSMP session were used as the baseline A1C and BMI values. For the 3- and 6-month data, the closest A1C and BMI values within 45 days of 3 or 6 months post-intervention were used. Additionally, we compared the percentage of patients who had undergone an eye exam or foot exam in the previous 12 months before the DSMP session or 12 months prior to their last primary care visit in the UC group, to the percentage of patients who received an eye or foot exam in the subsequent 12 month post intervention period.

#### Data analysis

A sample size of 343 patients per group was estimated as sufficient to test a difference in the mean change in A1C between the DSMP and UC groups assuming a two-sided test of significance, a mean standard deviation (SD) of DSMP A1C of 0.7 (SD 2.1) and UC A1C of 0.3 (SD 0.9), 90% power, and alpha = 0.05. Descriptive statistics were calculated for all variables. Continuous variables were compared by t-test, and categorical variables were compared by Chi-square test.

### Results

The DSMP (n = 513) and UC (n = 857) groups’ characteristics at baseline are summarized in Table 1. The mean (SD) age was significantly lower, though not considered to be clinically significant, in the DSMP group compared to the UC group: 61.3 (11.9) years vs. 66.5 (13.5) years, p < 0.001.

**Table 1:** T2D patient characteristics at baseline

	DSMP	UC	p value
n	513	857	
Males, n (%)	259 (50.5)	414 (48.3)	0.44
Age, mean (SD)	61.3 (11.9)	66.5 (13.4)	< 0.001
A1C, % mean (SD)	8.1 (1.8)	7.1 (1.2)	< 0.001
A1C, mmol/mol, mean (SD)	65.0 (19.7)	54.0 (13.1)	< 0.001
BMI, mean (SD)	32.1 (7.7)	30.6 (7.4)	0.001
Patients having had eye exams, n (%)	289 (56.3)	568 (66.3)	< 0.001
Patients having had foot exams, n (%)	261 (51.0)	436 (51.0)	0.999

*T2D* type 2 diabetes, *DSMP* diabetes self management program, *UC* usual care

The baseline A1C and BMI values of patients in the DSMP group were significantly higher compared to the UC group: A1C, 8.1% (1.8) vs. 7.1% (1.2), p < 0.001, and BMI, 32.1 kg/m^2^ (7.7) vs. 30.6 kg/m^2^ (7.4), p = 0.001, respectively. At baseline, a significantly larger proportion of patients in the UC group had undergone an eye exam in the past 12 months compared to the DSMP group: 66.3% vs. 56.3%, p < 0.001. The proportion of patients who had undergone a foot exam in the previous 12 months was equal in the UC and DSMP group: 51.0% vs. 51.0%, p = 0.999, respectively.

Among the patients in the DSMP group, 45.8% attended one or two group meetings and 13.2% attended three or four group meetings; the remaining 41% of the patients were seen individually. Of the patients who were seen individually, 77.7% had 1–2 individual appointments, 17.1% had three appointments, and 5% had 4–8 appointments. Of the DSMP-group patients, 55.8% received 1–3 phone calls, and a minority (17.7%) received ≥ 4 calls. On average, the total amount of contact time per patient (individual appointments plus group meetings) was 4 h.

At 3 months post-intervention, patients in the DSMP group had a significantly greater mean change in A1C; i.e., a mean (SD) − 0.8% (1.5) reduction compared to that of the UC group at − 0.2% (0.9), p < 0.001. (Table 2) The mean decrease in A1C at 6 months post-intervention was also significantly greater in the DSMP group at − 0.6% (1.3) compared to the UC group at − 0.2% (1.1), p < 0.001. There was no significant difference in the mean BMI change between the patients in the DSMP versus UC groups at 3 months: − 0.2 (1.4) and − 0.1 (1.4), respectively, p = 0.076 and at 6 months: − 0.2 (1.8) versus − 0.1 (1.9), respectively, p = 0.172 (Table 3).

**Table 2:** T2D A1C change 3 and 6 months post-intervention

A1C % change	DSMP		UC		p
	n	Mean (SD)	n	Mean (SD)	
3 months	295	− 0.8 (1.5)	857	− 0.2 (0.9)	< 0.001
6 months	234	− 0.6 (1.3)	475	− 0.2 (1.1)	< 0.001

*T2D* type 2 diabetes, *DSMP* diabetes self management program, *UC* usual care

**Table 3:** T2D BMI change 3 and 6 months post-intervention

BMI (kg/m^2^) change	DSMP		UC		p
	n	Mean (SD)	n	Mean (SD)	
3 months	382	− 0.2 (1.4)	857	− 0.1 (1.4)	0.076
6 months	311	− 0.2 (1.8)	713	− 0.1 (1.9)	0.172

*T2D* type 2 diabetes, *DSMP* diabetes self management program, *UC* usual care

Of the patients who had not undergone an eye exam in the previous 12 months (224 in DSMP group and 289 in UC group), the percentage undergoing an eye exam in the subsequent 12 months was not significantly different between groups [98 (44%) DSMP group vs. 136 (47%) in UC group, p = 0.456]. Similarly, of the patients who had not undergone a foot exam in the previous 12 months (252 in DSMP group and 421 in UC group), the percentage undergoing a foot exam in the subsequent 12 months was not significantly different between groups [72 (29%) DSMP group vs. 139 (33%) in UC group, p = 0.229].

### Discussion

Overall, at 3 months after the intervention, patients who participated in the short-term model of the DSMP achieved a significantly greater reduction in A1C compared to patients who received only UC. Moreover, the A1C values of the DSMP group continued to be significantly lower than those of the UC patients at 6 months post-intervention. Patients in the DSMP group had higher baseline A1C values than the UC group, as would be expected, since physicians would refer diabetes patients with poorer glycemic control to a DSMP. However, the magnitude of the A1C reduction in the patients who participated in the DSMP (− 0.8 at 3 months and − 0.6 at 6 months) compares favorably to a meta-analysis and a review in which the reported mean decrease in A1C in T2D patients who attended DSME programs was 0.74–0.76 at follow-up [3, 4].

A 2015 meta-analysis of 132 randomized, controlled trials of ≥ 4-week DSME programs published between 1993 and 2015 concluded that DSME behavioral programs that provide ≤ 10 h of education offer little benefit for A1C reduction [5]. However, our present findings demonstrated that 2–4 h of a concentrated, interactive DSME program over a period of 1–2 weeks with a telephone follow-up from a pharmacist diabetes educator within 1–2 weeks of the final group meeting or individual appointment significantly improved glycemic control within a short term in patients with T2D. While patients have provided many diverse and complex reasons for not attending DSME programs, one significant reason is logistical, including the length of a program [6]. The relatively short DSMP described herein enables pharmacist educators to cover several topics at the first group meeting or individual appointment and address the most concerning questions that patients have at their first visit. Moreover, the follow-up telephone calls enabled our pharmacist educators to address the individual needs of each patient and provide support after initially establishing a relationship with patients.

Improving glycemic control has been reported to cause weight gain, particularly with upward insulin titration [7, 8]. We observed no significant change in the patients’ BMI at 3 or 6 months following intervention in either the DSMP or UC group. While the DSMP discussed nutrition, physical activity and behavioral strategies to lose weight, weight loss was not observed in the patients; however, their BMI values did not increase. We also observed no significant difference between the percentage of patients who completed the DSMP and underwent eye and foot examinations within 12 months of their first group meeting or individual appointment compared to the UC group. The U.S. Centers for Disease Control and Prevention stated that nationwide in 2012, 64.9% of patients with diabetes had reported undergoing a dilated eye exam and 71.2% reported having completed an annual foot exam in the previous 12 months [9]. Both the present DSMP and UC groups had low foot examination rates.

### Limitations

The limitations of this study include the retrospective data collection at a single site and use of a non-randomized comparator group. Medical examinations (such as eye exams) and lab data that were not performed at a UCSDHS clinic were not available for review. In addition, the ability of the pharmacist educators to order diabetes testing supplies and refills of diabetes medications may have contributed to the favorable glycemic response observed in this study, since this ability likely facilitated self-monitored blood glucose testing and diabetes medication adherence. Another limitation is that although the pharmacist educators provided medication recommendations in the patients’ EMR, we did not measure the percentage of recommendations that were subsequently accepted by referring providers. Compared to the DSMP patients, more of the UC patients at baseline had undergone an eye exam in the previous 12 months, suggesting that patients who were less likely to see an eye specialist and/or less likely to adhere to an eye specialist’s regimen were being referred to the DSMP. Lastly, the effects on the patients’ A1C values beyond 6 months after completion of the DSMP were not evaluated in this study.

## Abbreviations

T2D: type 2 diabetes
DSME: diabetes self-management education
DSMP: diabetes self-management program
UC: usual care from the primary care physician
BMI: body mass index
UCSDHS: University of California San Diego Health System
EMR: electronic medical records
SD: standard deviation
